# Functional Characterization of AbeD, an RND-Type Membrane Transporter in Antimicrobial Resistance in *Acinetobacter baumannii*

**DOI:** 10.1371/journal.pone.0141314

**Published:** 2015-10-23

**Authors:** Vijaya Bharathi Srinivasan, Manjunath Venkataramaiah, Amitabha Mondal, Govindan Rajamohan

**Affiliations:** Council of Scientific Industrial Research- Institute of Microbial Technology, Sector 39 A, Chandigarh-160036, India; Université d'Auvergne Clermont 1, FRANCE

## Abstract

**Background:**

*Acinetobacter baumannii* is becoming an increasing menace in health care settings especially in the intensive care units due to its ability to withstand adverse environmental conditions and exhibit innate resistance to different classes of antibiotics. Here we describe the biological contributions of *abeD*, a novel membrane transporter in bacterial stress response and antimicrobial resistance in *A*. *baumannii*.

**Results:**

The *abeD* mutant displayed ~ 3.37 fold decreased survival and >5-fold reduced growth in hostile osmotic (0.25 M; NaCl) and oxidative (2.631 μM–6.574 μM; H_2_O_2_) stress conditions respectively. The *abeD* inactivated cells displayed increased susceptibility to ceftriaxone, gentamicin, rifampicin and tobramycin (~ 4.0 fold). The mutant displayed increased sensitivity to the hospital-based disinfectant benzalkonium chloride (~3.18-fold). In *Caenorhabditis elegans* model, the *abeD* mutant exhibited (P<0.01) lower virulence capability. Binding of SoxR on the regulatory fragments of *abeD* provide strong evidence for the involvement of SoxR system in regulating the expression of *abeD* in *A*. *baumannii*.

**Conclusion:**

This study demonstrates the contributions of membrane transporter AbeD in bacterial physiology, stress response and antimicrobial resistance in *A*. *baumannii* for the first time.

## Introduction

*Acinetobacter baumannii* is a nosocomial pathogen that causes a wide range of clinical illnesses in immunocompromised patients including bacteremia, pneumonia, meningitis, urinary tract, wound and skin infections [1]. Overall mortality rates for nosocomial *Acinetobacter* infection have been reported to range from 19 to 54% [2, 3]. A report from India suggests that around ~ 10% of all hospital-acquired infections were caused by *A*. *baumannii* [4, 5]. The pathogen has attracted the attention of researchers globally as it has the ability to attain antibiotic resistance genes, resist desiccation and stay on abiotic surfaces for long [1]. Though *A*. *baumannii* has the ability to acquire additional resistance elements, overproduction of membrane transporters having wide spectrum antibiotic preference is also well correlated with the multidrug resistance (MDR) phenotype displayed by this notorious human pathogen [6, 7]. The efflux pumps in prokaryotic kingdom are structurally diverse and belong to one of the five different super families: major facilitator super family (MFS), multiple antimicrobial and toxic compounds extrusion family (MATE), small multidrug resistance family (SMR) ATP-binding cassette family (ABC) and resistance nodulation cell division family (RND). Among these, the RND-type efflux pump is the predominant one known to be involved in antibiotic resistance across Gram-negative bacteria [8, 9, 10].

The first characterized efflux pump AdeABC is regulated by AdeRS and Magnet *et al* has demonstrated its specificity to different classes of antibiotics [11, 12]. Damier *et al* identified the AdeN modulated intrinsic tripartite efflux pump AdeIJK and demonstrated its functions in conferring broad-spectrum antimicrobial resistance [13, 14]. Coyne *et al* demonstrated functions of LysR regulated AdeFGH in mediating increased resistance to diverse substrates [15]. The clinical isolate *A*. *baumannii* AYE strain (Genbank accession number: NC_010401, 4 Mb, 3712 proteins, GC: 39.3% with 86Kb resistance island) was involved in national level outbreak in France in 2001 and is known to harbor several genes annotated as putative efflux pumps [16]. Our subsequent analysis revealed the presence of an uncharacterized open reading frame [lacking cognate membrane fusion protein (MFP) and outer membrane protein (OMP)] that exhibited identity to AcrD-like transporter genes. Here, we have elucidated the unprecedented physiological contributions of the *acrD* homolog (designated as *abeD*) and its regulation in *A*. *baumannii* for the first time.

## Materials and Methods

### Bacterial strains, media and methods

*A*. *baumannii* AYE strain was procured from ATCC. *E*. *coli* KAM32, a highly susceptible strain that lacks major multidrug efflux pumps (*acrAB* and *ydhE*) was used as a host for heterologous studies (kindly provided as a gift by Dr. Tomofusa Tsuchiya). The Luria-Bertani (LB) broth or LB agar (Difco, Becton-Dickinson, Sparks, MD) was used and 400 mg/L hygromycin or 200 mg/L zeocin was added when required. Standard techniques were performed following manufacturer’s instruction or as described previously [17, 18, 19]. Custom-synthesized primers were used in this study (Eurofins MWG operons, Germany).

### Generation of Δ*abeD* and Δ*abeD*Ω*abeD* constructs in *A*. *baumannii*

The putative *acrD* homolog, ABAYE0827 (designated *abeD*) spans from nucleotides 884427 to 887522 bps (*abeD*: 3096bp, 1031aa, 113Kda) in genome sequence of *A*. *baumannii* AYE. A 930bp partial gene was amplified by PCR using Δ*abeD*-F/Δ*abeD*-R primers (Table 1) and cloned into hygromycin cassette containing pUC4K derived suicide vector. The recombinant construct pUC-*abeD* was electroporated into *A*. *baumannii*, and disruption was confirmed by Southern and sequencing analysis, the obtained strain was denoted as Δ*abeD*. With zeo-NT and zeo-CT primers (Table 1), the zeocin resistance marker was amplified from pCR Blunt II-TOPO vector (Life Technologies) and cloned into the shuttle vector pWH1266. Using FL*abeD*-F and FL*abeD*-R primers (Table 1), the full-length transporter gene along with its promoter was cloned in the modified pWH1266. The generated construct was electroporated into Δ*abeD* to obtain the complemented strain Δ*abeD*Ω*abeD*.

**Table 1:** Primers used in this study.

Primer name	Primer sequences (5’-3’)
Δ*abeD*-F	ATTCAGACCTCTAAGCTCATCCACCAAACT
Δ*abeD* -R	AAAGTTTTCGAAAGTTGAATAATTTTTACT
FL*abeD* -F	GACTCAAGTTTCGGTGCTTTGTTTGACATGATCATGAAGAATT
FL*abeD*-R	GAAAAGTTAATCTTAATATACTTGATTTGATGCCCGTTTT
zeo-NT	ACATTCAAATATGTATCCGCTCATGAGACAATAAC
zeo-CT	TCAGTCCTGCTCCTCGGCCACGAAGTGCACGC
AB2390-NT	TATACATATGAGTGAAAGGAATCAAAGTCGTTTG
AB2390-CT	TCTAGGATCCCTATGGTCTTTCTAAAATACGTGG
prom*abeD*-F	GGCTGCTCGGTATGATGTCGTTAATTAACA
prom*abeD*-R	TGCTTTGTTTGACATGATCATGAAGAATTC
AB0827RTF	ATTGGTTTAGCGTGGACAGG
AB0827RTR	ATAGATGTCATTACGGCGGC
RTrpoBNT	GCGGTTGGTCGTATGAAGTT
RTrpoBCT	TGGCGTTGATCATATCCTGA

### Bacterial growth curves

The growth profile of WT (control strain: *A*. *baumannii*, AYE), Δ*abeD* and Δ*abeD*Ω*abeD* were examined in LB broth having different pH [17, 20]. Absorbance was measured continuously for 10h at an interval of every 30 mins at OD_600nm_ using Bioscreen C automated growth analysis system (Labsystems, Helsinki, Finland). Every time the media was prepared freshly and independent experiments were carried out and the average of such three experiments is plotted here.

### Growth inhibition assay

The growth inhibition assay to identify the occurrence of active efflux was performed as mentioned before with slight modifications [17, 20]. The WT, Δ*abeD* and Δ*abeD*Ω*abeD* cultures at OD_600nm_ = 0.1 were inoculated into LB medium with gentamicin (16.0 mg/L) in different experiments either alone or with efflux pump inhibitor phenylalanine arginyl β-naphthylamide; PAβN at 25mg/L or 50mg/L (Sigma, St. Louis, MO). The growth of WT, Δ*abeD* and Δ*abeD*Ω*abeD* was measured at OD_600nm_ at 37°C periodically in Bioscreen C automated growth analysis system. Other inhibitors carbonyl cyanide 3-chlorophenylhydrazone; CCCP 2.5 mg/L, 2, 4, Dinitrophenol DNP 2.5 mg/L; verampamil; VER 2.5 mg/L, reserpine; RES 2.5 mg/L (Sigma, St. Louis, MO) were also used in our study in independent experiments. The experiments were performed at least six times.

### Assays to measure motility behavior and biofilm forming ability

For motility assay, the *A*. *baumannii* cultures grown to OD_600nm_ = 1.0 was spotted in the center of the plate having different agar concentrations and incubated for 10h at 37°C [17, 20]. Upon their growth in an outward manner they formed a halo and the diameter indicated their extent of motility. Crystal violet binding assay to estimate biofilm forming ability was performed as described before [17, 20]. Cultures grown to OD_600nm_ = 0.1 was inoculated into LB broth and incubated at 37°C shaking for 24 hours. The biofilm was stained and reading was taken at OD_570nm_ using Synergy H1 Hybrid microplate reader (BioTek Instruments Inc., Winooski VT).

### Oxidative and nitrosative challenge assays

As to test the role of *abeD* in oxidative stress tolerance, disc assay was performed in which paper disks were treated with various amount of hydrogen peroxide (H_2_O_2_), air dried as reported before [21, 22]. The WT, Δ*abeD* and Δ*abeD*Ω*abeD* were grown to OD_600nm_ = 0.1 and plated on an agar plate. The paper disks were placed at the center and incubated at 37°C, the zone of inhibition was measured after 16h. Growth capabilities of WT, Δ*abeD* and Δ*abeD*Ω*abeD* in presence of different amounts of H_2_O_2_ were monitored and compared with WT by measuring the absorbance at OD_600nm_ periodically in Bioscreen C automated growth analysis system [17]. The survival ability of WT, Δ*abeD* and Δ*abeD*Ω*abeD* to different oxidative challenges was tested as described before [17]. Acidified sodium nitrite and sodium nitroprusside (SNP) were used to generate nitrosative stress and ability of WT, Δ*abeD* and Δ*abeD*Ω*abeD* to withstand the stress of these NO-releasing agents were tested as reported before [17, 23].

### Various stress challenge assays

The survival capability of WT, Δ*abeD* and Δ*abeD*Ω*abeD* to different stress conditions at various concentrations was performed as mentioned before [17, 20]. In this study wide range of substrates were tested for *e*.*g*. sodium chloride (NaCl), bile salt deoxycholate, antibiotics (gentamicin, kanamycin, neomycin), efflux pump substrates (ethidium bromide (EtBr), acriflavine, saffranine) and disinfectants (benzalkonium chloride, chlorhexidine and triclosan). All experiments were carried out independently for three times.

### Antibiotic susceptibility testing and OMP preparation

Analysis of antibiotic susceptibilities for WT, Δ*abeD* and Δ*abeD*Ω*abeD* was examined using commercial discs (Hi-Media, Bombay, India) as described previously following Clinical and Laboratory Standards Institute (CLSI) guidelines [17, 20]. The antibiotics tested were amikacin: AMK (10 mg/L), ampicillin: AMX (30 mg/L), ceftazidime: CAZ (30 mg/L), chloramphenicol: CHL (30 mg/L), clindamycin: CLD (10 mg/L), colistin: CST (10 mg/L), ceftriaxone: CTR (10 mg/L), gentamicin: GEN (30 mg/L), kanamycin: KAN (30 mg/L), methicillin: MET (30 mg/L), minocycline: MIN (10 mg/L), meropenem: MRP (10 mg/L), nalidixic acid: NAL (30 mg/L), neomycin: NEO (30 mg/L), ofloxacin: OFL (10 mg/L), oxacillin: OXA (10 mg/L), piperacillin: PIP (10 mg/L), polymyxin B: PMB (300 mg/L), rifampicin: RIF (30 mg/L), spectinomycin: SPT (10 mg/L), sparfloxacin: SPX (30 mg/L), tetracycline: TET (30 mg/L), ticarcillin: TIC (75 mg/L), tobramycin: TOB (30 mg/L) and vancomycin: VAN (10 mg/L). The minimum inhibitory concentration (MIC) for different antibiotics was determined using either E-strips or spotting on agar plates with different antibiotic concentrations as described before [17]. The OMPs from WT, Δ*abeD* and Δ*abeD*Ω*abeD* were prepared following procedure as described previously [17].

### Fluorimetric efflux studies

The fluorimetric EtBr assay was carried out as mentioned briefly. The cultures were grown to OD_600nm_ = 0.6 and pelleted. Further it was washed in PBS and resuspended to OD_600nm_ = 0.3. Glucose was added to the cellular suspension to a final concentration of 0.4% (v/v) and aliquots of 0.095 ml were transferred to 96 well plate. EtBr was added in aliquots of 0.005 ml to obtain final concentrations that ranged from 1.0 to 6.0mg/L. The fluorescence at emission 600nm/excitation 530nm was monitored for one hour in Synergy H1 Hybrid microplate reader and automatically recorded for each well after every 1 min at 37°C under the conditions described above. The effect of inhibitor CCCP in the accumulation of EtBr was determined under conditions that optimize efflux (presence of glucose and incubation at 37°C). The experiment was performed with the freshly autoclaved medium in triplicates at least six independent times before plotting the graph.

### Cloning of SoxR regulator and Gel shift assays

The *A*. *baumannii* strain AYE (4 Mb, 3712 proteins, GC: 39.3% with 86Kb resistance island) has ~ 214 signaling proteins in its genome www.mistb.com and functions of only few have been elucidated so far [16]. The MerR type DNA-binding HTH-type transcriptional regulator ABAYE2390 (*soxR*; 453bp, 150aa and 17.01 kDa) was cloned and expressed in our lab in our previous study. The purified protein and the radiolabelled *abeD* promoter fragment was used in gel shift assay. The binding specificity was confirmed using appropriate controls as described previously [17, 20].

### RNA isolation and real-time reverse transcription PCR (RT-PCR)

Total RNA was extracted from the log-phase cultures using the RNeasy Mini Kit (Qiagen) according to the manufacturer's instructions. The RNA isolation was done two independent times, quantified and PCR reactions with Maxima SYBR Green qPCR master mix (Fermentas) were performed using gene specific primers [17]. In the RT-PCR analysis *rpoB* (RTrpoBNT/CT) was used as an internal control and the specific amplification was confirmed by melting curve analysis. The relative gene expression was calculated using the 2^−ΔΔCt^ method [24], and the experiments were performed more than three times.

### Caenorhabditis elegans killing assay

Bacterial virulence assays were performed using nematode model, *C*. *elegans* strain Bristol N2 as reported previously [25]. To examine the ability of WT, Δ*abeD*, Δ*abeD*Ω*abeD* and *E*. *coli* OP50 strains to kill *C*. *elegans*, bacterial lawns of *A*. *baumannii* and *E*. *coli* control strain were prepared on nematode growth (NG) media and incubated at 37°C for 6h. The plates were kept at room temperature for 1hr and then seeded with L4-stage worms (25 to 30 per plate). Further, the seeded plates were incubated at 25°C and examined for live worms under a stereomicroscope (Leica MS5) after every 24 hours. When the worm did not react to touch, it was considered dead. For each strain, experiments were performed three times with five replicates respectively.

### Cloning of *abeD* in *E*. *coli* KAM32 for heterologous studies

The uncharacterized *abeD* homolog was amplified using primer pairs FL*abeD*-F/FL*abeD*-R, and cloned into PCR-XL-TOPO vector (Invitogen) following standard procedures. The obtained recombinant construct *pabeD* was transformed into *E*. *coli* KAM32 to obtain *E*. *coli* KAM32/p*abeD* which was further used for antibiotic susceptibility, oxidative disc and fluorimetric assays for functional characterization.

### Bioinformatic analysis and Statistical analysis

To perform the homology searches, similarity and identity analysis, conserved domain architecture analysis, NCBI internet server was used. Data are shown as an average ± the standard error. Standard deviations were calculated using Microsoft-Excel. On raw data, statistical analysis was done with the help of paired Student’s t-test. P values of <0.05 were considered significant.

## Results

### *In silico* analysis of RND type membrane transporter AbeD

**Fig 1:**
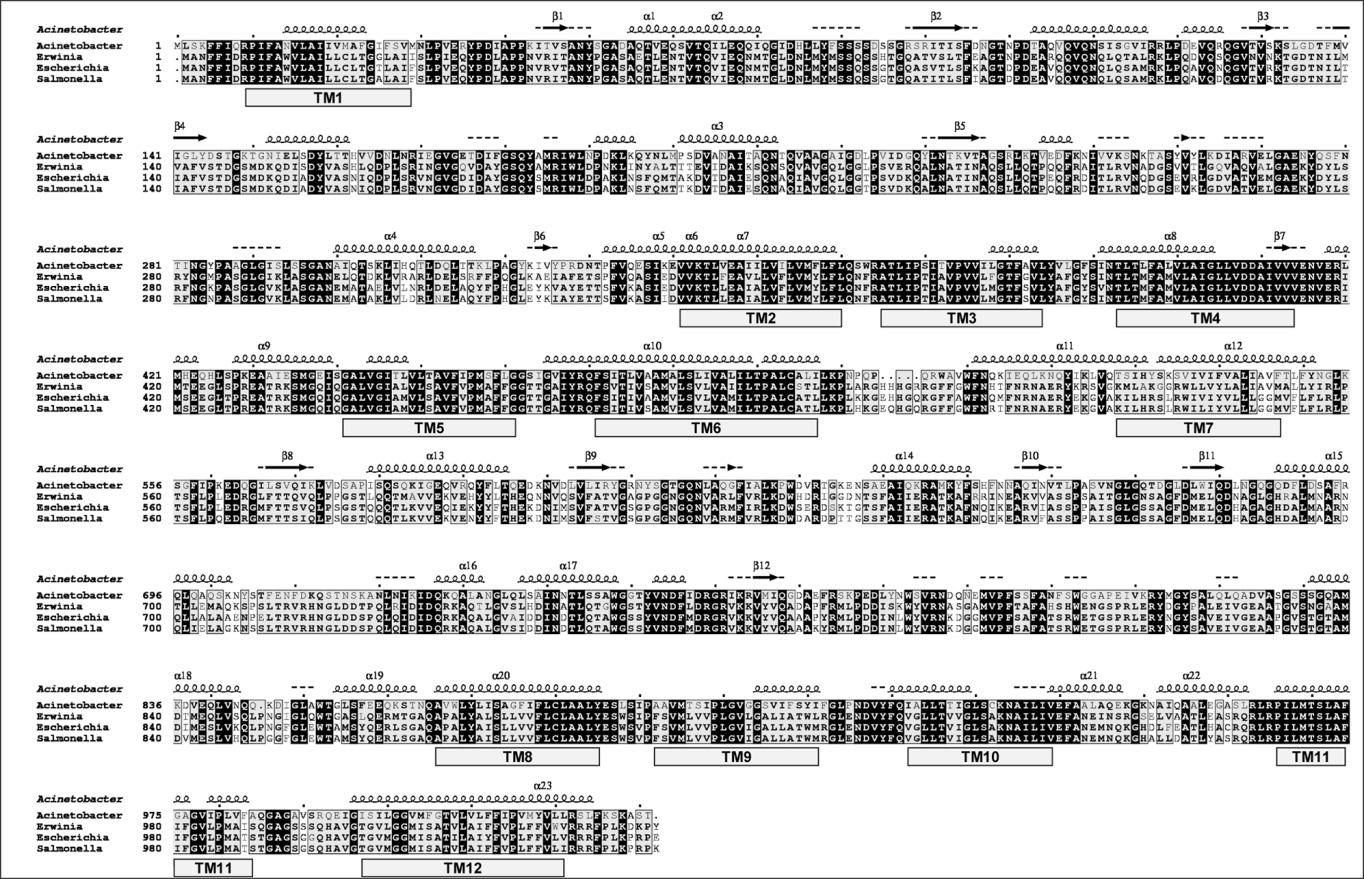
Alignment of multiple AbeD sequences and its homologs. Sequence alignments were made in CLUSTAL Omega (https://www.ebi.ac.uk/Tools/msa/clustalo) and formatting using the ESPript server (http://espript.ibcp.fr/ESPript/cgi-bin/ESPript.cgi). The accession numbers of AbeD from *A*. *baumannii* is CAM85780.1, *Erwinia amylovora* ATCC 49946 is YP_003539485.1, *Escherichia coli* is YP_490697.1, and *Salmonella enterica* serovar Typhimurium is ADX18241.1. The predicted secondary structural elements of *A*. *baumannii* AbeD are shown on the lines above the sequence alignment using PSIPRED (http://bioinf.cs.ucl.ac.uk/psipred). Transmembrane regions TM1 to TM12 were predicted using HMMTOP (http://www.enzim.hu/hmmtop/index.php). The arrows indicate β-sheet and the coils indicate α-helices respectively. Residues strictly conserved have a black background and is indicated by bold letters; residues conserved between groups are boxed.

The transporter AbeD is a 1031 aa long protein, predicted to be a member of Gram-negative bacterial hydrophobe/amphiphile efflux-1 family that belongs to RND superfamily. The sequence alignment of AbeD displays 47.1% identity and 68.5% similarity to the *E*.*coli* AcrD protein, 47.6% identity and 68.8% similarity with AcrD of *Salmonella enterica* subsp. *enterica* serovar Typhimurium, 47.7% identity and 69.5% similarity with AcrD of *Erwinia amylovora*, 53.6% identity and 74.8% similarity with AcrD of *Stenotrophomonas maltophilia* (YP_002027309.1), ~ 53.6 to ~ 54.9% identity and ~ 74.9% similarity with AcrD of *A*.*venetianus* (ENV36179.1), *A*. *oleivorans* (ADI89711.1), *A*. *nosocomialis* (ERL67989.1), *A*. *guillouiae* (ENU59765.1), 61.4% identity and 80.5% similarity with AcrD of *Acinetobacter* sp. ADP1 (ACIAD2836) [Fig 1]. A phylogenetic tree was constructed using AbeD and other characterized members of *Enterobacteriaceae* and AbeD formed a separate cluster, indicating that it’s divergent from its homologs (data not shown). The AbeD protein is conserved (100% identity) across different *A*. *baumannii* strains (YP_002324730.1, YP_002320396.1, EJO40799.1, EKL39351.1 and EKP54977.1). The clustal comparison of proteins exhibited that they were well conserved across *Acinetobacters*. Further analysis of AbeD revealed the presence of β-sheet, α-helical domains, and 12 transmembrane domains. Interestingly, the essential charged residues in *E*.*coli* AcrB in the transmembrane domain, D408, D409, E415 (in TM4), K933 (in TM10) and T970 (in TM11) are well conserved in AbeD, which are responsible for proton relay pathway / transport. Overall, *in silico* analysis suggests that AbeD is indeed an putative transporter protein that belongs to the RND-type and its characterization would shed let light on its biological relevance.

### Characterization of AbeD in heterologous host

Here, we deciphered the functions of AbeD in *E*. *coli* KAM32. The antibiotic susceptibilities indicated that *abeD* does confer drug resistance in heterologous host [S1A Fig], but to get a clearer picture, the precise MICs were determined. The *abeD*—transformed cells (*E*. *coli* KAM32/p*abeD*) displayed higher (fold increase in brackets) MIC for erythromycin (4-fold), ertapenem (5-fold), and amikacin (2.7-fold) [Table 2]. Results obtained from oxidative disc assay indicated the importance of AbeD transporter in conferring tolerance against oxidative stress [S1B Fig]. The fluorimetric efflux assay using EtBr as a substrate confirmed the functions of AbeD in active efflux mechanism [S1C and S1D Fig]. To elucidate the functions of membrane transporter in native host, the gene was inactivated and various experiments were performed to delineate its physiological relevance by using *A*. *baumannii* AYE as the model strain.

**Table 2:** Determination of MIC for KAM32/p*abeD* and KAM32/vector.

Antibiotics	KAM32/vector	KAM32/p*abeD*	Fold change ^a^
Amikacin	0.75	2	2.66
Amoxycillin/Sulbactam	1	2	2
Ceftazidime	0.25	0.5	2
Clindamycin	0.75	1.5	2
Erythromycin	2	8	4
Ertapenem	0.012	0.064	5.33
Levofloxacin	0.023	0.058	2.51
Meropenem	0.125	0.25	2
Ticarcillin	2	6	3
Trimethoprim	0.032	0.064	2

E-strips were used to determine the precise MIC for different group of antibiotics such as Amikacin, Amoxycillin/Sulbactam, Ceftazidime, Clindamycin, Erythromycin, Ertapenem, Levofloxacin, Meropenem, Ticarcillin and Trimethoprim following the CLSI guidelines.

### Novel contributions of AbeD in general physiology in *A*. *baumannii*

**Fig 2:**
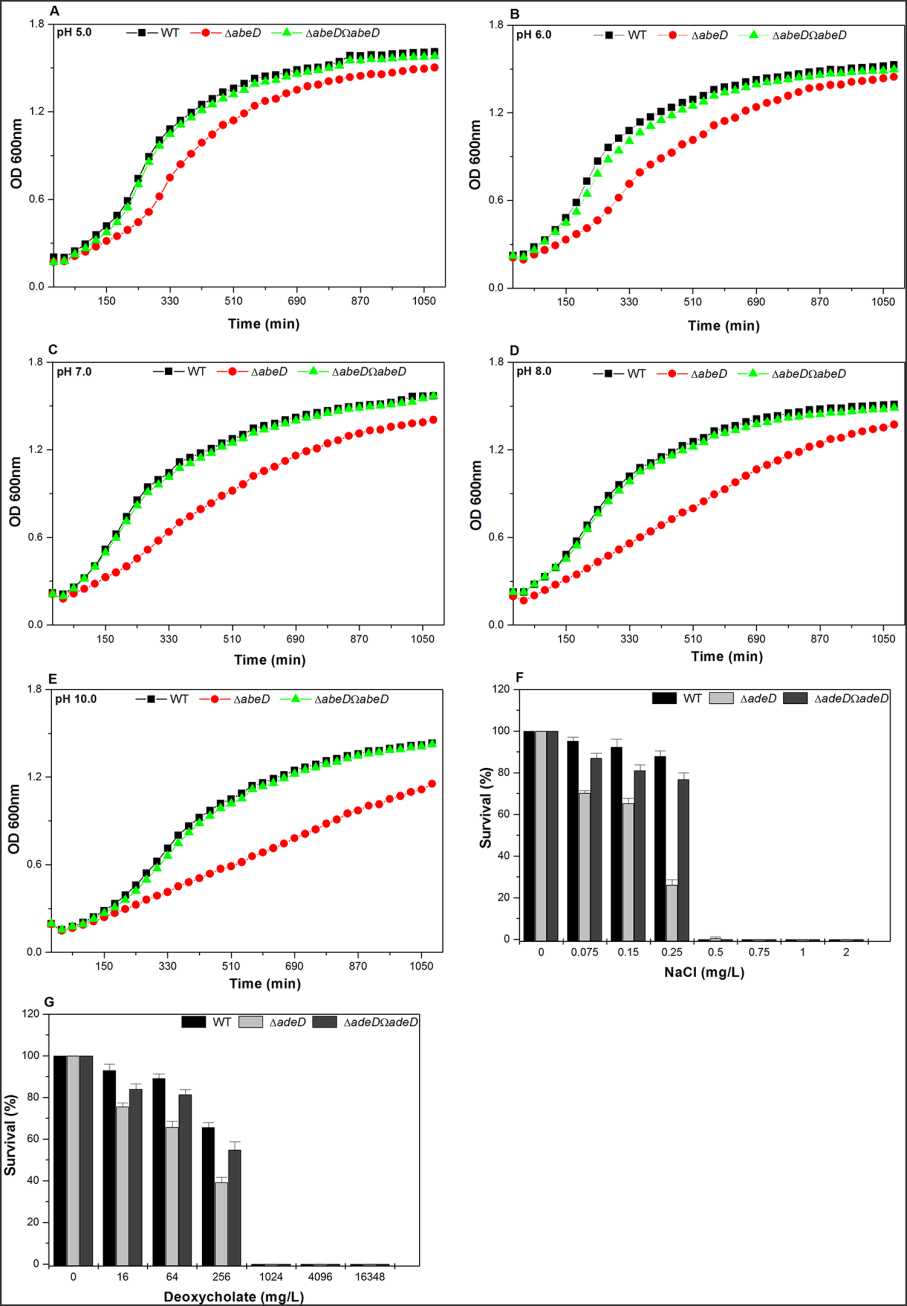
Impact of *abeD* disruption on growth and physiology in *A*. *baumannii*. The influence of AbeD on growth of bacteria was monitored in WT, Δ*abeD* and Δ*abeD*Ω*abeD* in LB medium at pH (5.0 (A), 6.0 (B), 7.0 (C), 8.0 (D), 10.0 (E). The average of independent experiments done three times is used to plot the graph. The survival capability of native strain to grow in the presence of NaCl was monitored and it was higher when compared to Δ*abeD* regardless of the inoculum size (F). The capacity of WT to grow in the presence of deoxycholate was compared to Δ*abeD*, the completely transcomplemented Δ*abeD*Ω*abeD* strain restored the ability to tolerate the stress (G).

Southern analysis and DNA sequencing confirmed the inactivation of *abeD* in *A*. *baumannii* AYE [S2 Fig] and the constructs were further used for functional characterization. Analysis of growth profiles in LB at different pH revealed that *abeD* mutant exhibited 1.62-fold ± 0.0215 (at pH 5.0) [Fig 2A], 1.65-fold ± 0.06 (at pH 6.0) [Fig 2B], 1.72-fold ± 0.036 (at pH 7.0) [Fig 2C], 1.85-fold ± 0.057 (at pH 8.0) [Fig 2D] and 1.6 fold ± 0.047 (at pH 10.0) [Fig 2E] stunted growth compared to WT strain respectively (P<0.001). Overall results demonstrated that loss of *abeD* did affect the growth capability in *A*. *baumannii*. Secondly, on spotting the cultures on plates with different agar concentrations, the WT profusely grew outwardly from the point of inoculation while the mutant could not; clearly indicating the loss in motility behavior [S3A Fig]. Thirdly the adherence property of strains was tested by measuring their ability to form biofilm and we found Δ*abeD* exhibited only ~ 1.2-fold ± 0.047 lesser ability compared to WT strain [S3B Fig] which tells that role of AbeD in this phenomenon was merely marginal. The capability of WT, Δ*abeD* and Δ*abeD*Ω*abeD* to grow under different osmotic stress challenges were tested, and in the presence of 0.25M NaCl, survival ability of WT was ~ 3.37 fold ±0.017 higher in comparison to Δ*abeD* irrespective of the starting inoculum of the culture (P = 0.0119) [Fig 2F]. When tested with sodium deoxycholate (bile salt), for *e*.*g*. in the presence of 256 mg/L of salt, survival ability of WT was ~ 1.67 fold higher (±0.022) compared to Δ*abeD* (P = 0.005) [Fig 2G]. Conclusively, we found *abeD* disruption affected bacterial growth and ability to tolerate gastrointestinal-like stress challenges.

### Functions of AbeD in oxidative and nitrosative stress tolerance in *A*. *baumannii*

**Fig 3:**
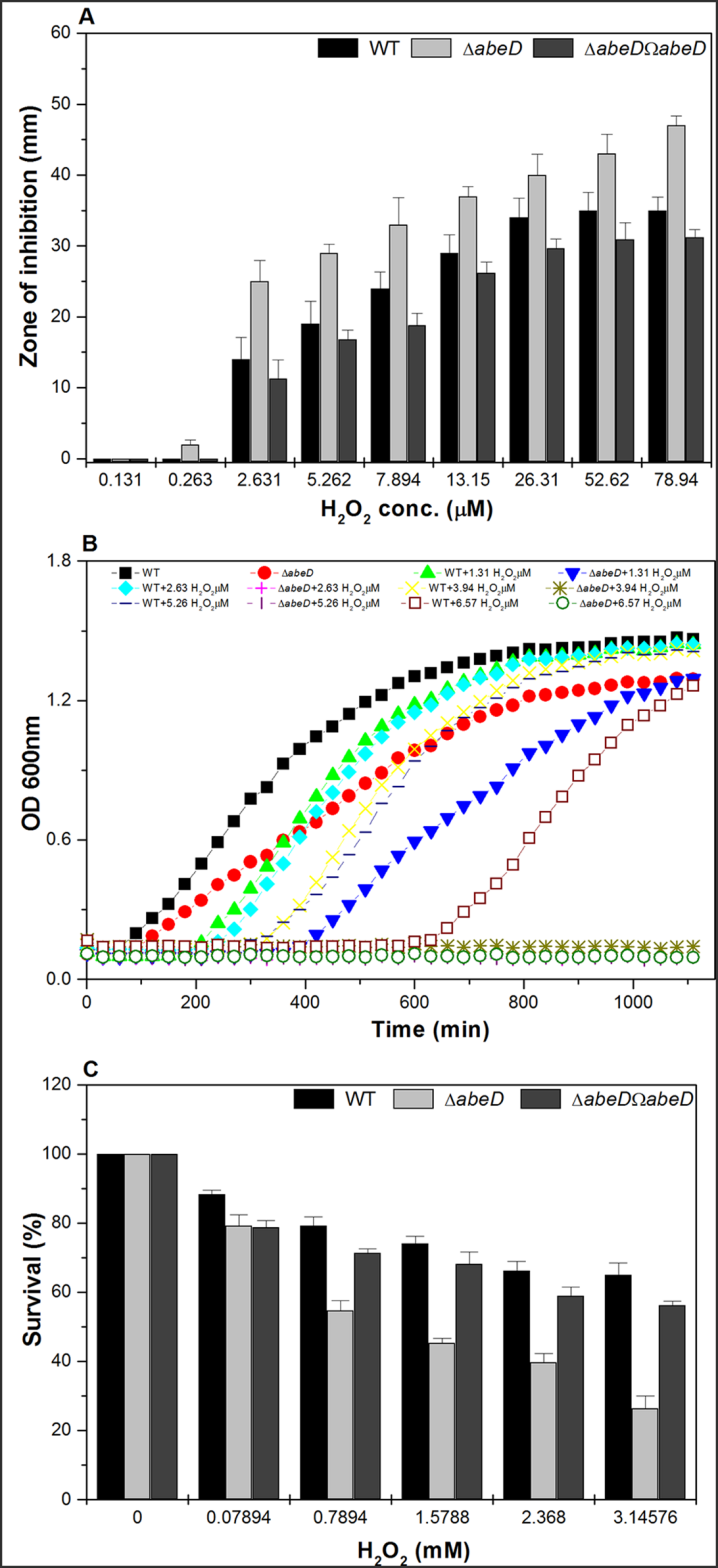
Hydrogen peroxide stress assays. A, The behavior of WT, Δ*abeD* and Δ*abeD*Ω*abeD* strains. The ability of WT, Δ*abeD* to resist various levels of H_2_O_2_ was tabulated performing disc diffusion assay and Δ*abeD* remained sensitive to the challenges. B, The impact of various concentrations of H_2_O_2_ on the growth ability of WT and Δ*abeD*. The differences between WT and Δ*abeD* are statistically significant (P < 0.05) for all different H_2_O_2_ concentrations used. C, The survival ability of WT and Δ*abeD* towards varied concentrations of H_2_O_2_ was elucidated as described previously. The differences between WT and Δ*abeD* are statistically significant (P < 0.05) for all concentrations used.

The H_2_O_2_ disc assay showed that the *abeD* mutant at 2.631μM was 1.78-fold and 5.262 μM was 1.52-fold sensitive than the wild-type (P = 0.00467) [Fig 3A]. The *abeD* mutant displayed 9.67-fold (P = 0.0003) [mean of fold differences of growth for WT/mutant for 18h], 5.49-fold (P = 0.0002), 8.52-fold (P = 0.0005), 4.24-fold (P = 0.0006) decreased growth with respect to WT in LB in presence of 2.631 μM, 3.9465 μM, 5.262 μM and 6.5775 μM concentrations of H_2_O_2_ respectively [Fig 3B]. In oxidative survival assay, the *abeD* mutant exhibited 1.6-fold (P = 0.0011) and 2.4-fold (P = 0.00032) reduced survival compared to WT strain when exposed to 2.3682 mM and 3.1576 mM concentrations of H_2_O_2_ respectively [Fig 3C]. The contribution of *abeD* in mediating protection against nitrosative stress response was tested similarly as mentioned above and we found that the ability of WT, Δ*abeD* and Δ*abeD*Ω*abeD* to grow in LB broth containing SNP and nitrite in different concentrations at pH 7.0 and 6.0 was only marginally affected. Survival assay using SNP also reconfirmed our observations [data not shown]. Therefore, results depict the crucial contributions of AbeD in salvaging against oxidative assails in *A*. *baumannii* for the very first time.

### Prominent functions of AbeD in mediating antimicrobial resistance in *A*. *baumannii*

**Fig 4:**
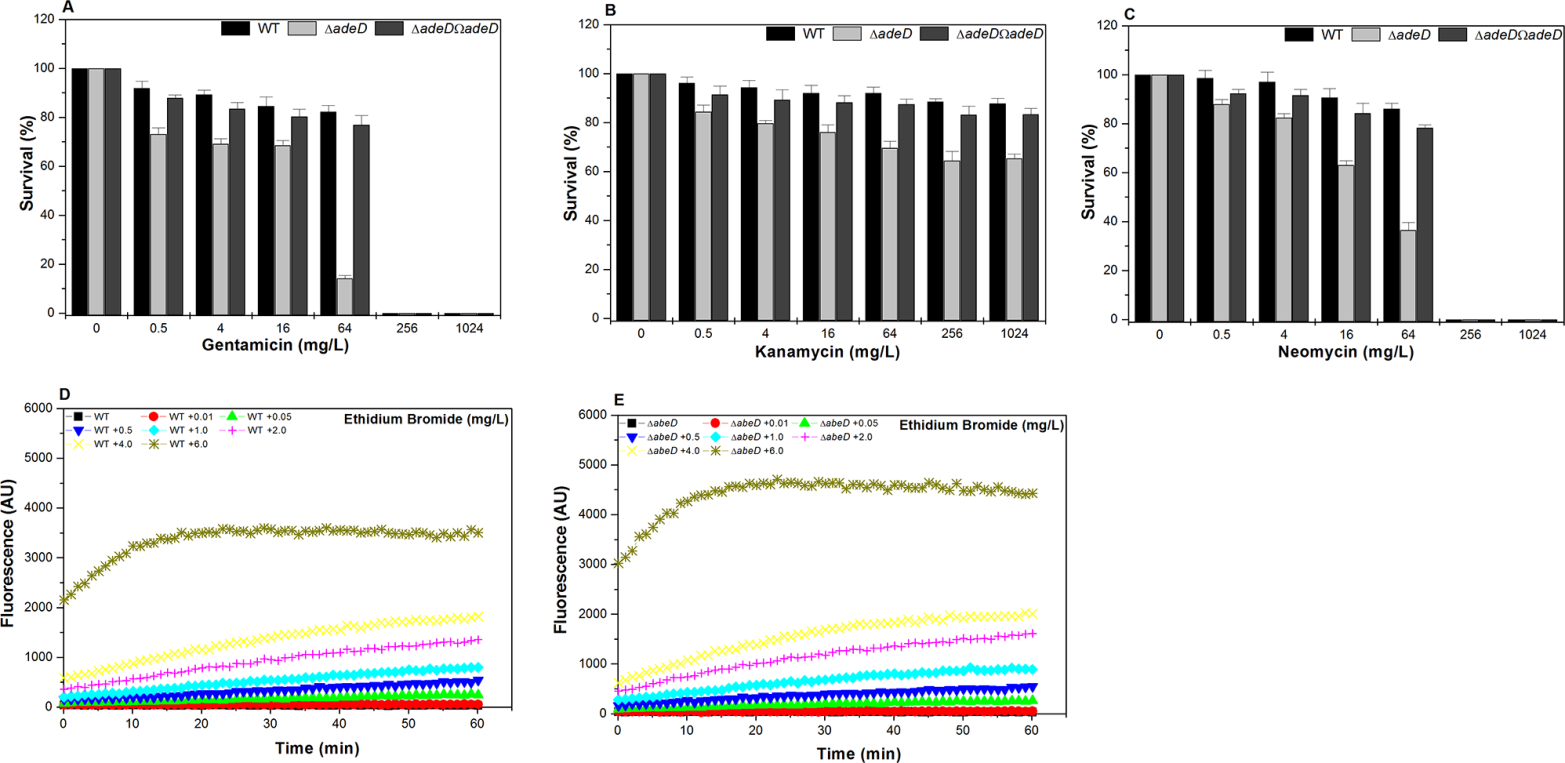
Contributions of AbeD in antibiotic resistance in *A*. *baumannii*. Survival assays using gentamicin (A), kanamycin (B), neomycin (C) are shown in bar graphs. Using the *A*. *baumannii* strains WT (D) and Δ*abeD* (E) cells EtBr accumulation assay at final concentrations that ranged from 0.01 mg/L to 6.0 mg/L was tested as described in methods section. The fluorescence was monitored in spectrofluorometer (Hitachi) at 37°C.

The study illustrated that Δ*abeD* showed increased susceptibility towards ceftriaxone, gentamicin, rifampicin, and tobramycin [Table 3]. The survival ability of WT, Δ*abeD* and Δ*abeD*Ω*abeD* to different classes of antibiotics were tested, and WT displayed higher survival ability compared to mutant *e*.*g*. gentamicin (P = 0.010, Fig 4A), kanamycin (P = 0.002, Fig 4B) and neomycin (P = 0.0016, Fig 4C). Interestingly, the total CFU count of WT at 64 mg/L of gentamicin and neomycin was 5.825-fold ± 0.058 and 2.361-fold ± 0.078 higher than Δ*abeD* cells respectively. Overall, results demonstrate that *abeD* is a novel multidrug resistance determinant in *A*. *baumannii*. Similarly, the *abeD* mutant cells showed decreased survival ability when exposed to various efflux based substrates such as EtBr (P = 0.0201, S4A Fig), acriflavine (P = 0.034, S4B Fig), safranin (P = 0.0014, S4C Fig) indicating AbeD transporter to have broad substrate specificity.

**Table 3:** MIC determination for different antibiotics using WT, Δ*abeD* and Δ*abeD*Ω*abeD*.

Antibiotics	WT	Δ*abeD*	Fold change ^a^	Δ*abeD*Ω*abeD*
Amikacin	256	128	2	256
Cefepime	256	128	2	256
Ceftriaxone	256	64	4	256
Ertapenem	>32	16	2	>32
Erythromycin	16	4	4	16
Gentamicin	128	32	4	128
Neomycin	60	30	2	60
Norfloxacin	240	120	2	240
Piperacillin	120	60	2	120
Rifampicin	4	1	4	3
Tobramycin	10	2	5	8
Vancomycin	8	4	2	8

E-strips were used to determine the precise MIC for different group of antibiotics such as amikacin, cefepime, ceftriaxone, ertapenem, erythromycin, gentamicin, neomycin, norfloxacin, piperacillin, rifampicin, tobramycin and vancomycin following the CLSI guidelines. Units for MIC values are mg/L and only few representative drugs are shown here.

^a^ Fold change is the ratio of MICs for WT and Δ*abeD*.

### Impact on active efflux capability in *abeD* isogenic mutant in *A*. *baumannii*

Growth inhibition assay using different substrates and efflux pump inhibitor were performed for *e*.*g*. gentamicin; 16.0 mg/L, with PAβN 25 mg/L [S4D Fig] or PAβN 50 mg/L [S4E Fig]; and kanamycin [data not shown] which pinpointed lower growth by mutant indicating diminished efflux capacity in the disrupted construct. To verify the crucial role of AbeD in active efflux, cells were processed as described before to decipher the difference in accumulation ability between WT and Δ*abeD* using the efflux substrate EtBr. As the mutant lacks functional AbeD transporter, the degree of fluorescence was lower in WT [Fig 4D] relative to mutant [Fig 4E]. We also observed variations in OMP profile indicating expression of additional proteins for *e*.*g*. ~25 kDa, ~35 kDa and ~40 kDa possibly to oppose MDR stress in Δ*abeD* (data not shown). Overall results converge to say that AbeD is involved in conferring MDR exploiting active efflux mechanism.

### Disinfectant challenge assays using WT and *abeD* isogenic mutant

Assays using WT, *abeD* and *abeD*Ω*abeD* in presence of varied concentrations of benzalkonium chloride [P = 0.025] [S5A Fig], chlorhexidine [P = 0.017] [S5B Fig] and triclosan [P = 0.0259] [S5C Fig] and growth inhibition assay with chlorhexidine [data not shown] authenticated the ability of AbeD in conferring biocide resistance in *A*. *baumannii*. So far data discussed in this report demonstrate that AbeD possess wide substrate preference and confers antimicrobial resistance by active efflux mechanism in *A*. *baumannii*.

### Expression analysis in *A*. *baumannii*

The expression of *abeD* was deciphered in different clinical strains from our collection. (Strains were randomly collected, from different Medical centers in India, isolated during 2012–2014; identified by *gyrA* sequencing; extremely MDR; diversity of resistome identified; manuscript under preparation). The expression of *abeD* relative to the sensitive strain *A*. *baumannii* SDF, was checked in *A*. *baumannii* AYE and 10 different MDR strains and we found an increased expression of *abeD* transporter (4.92 to 12.13 ± 2.21 fold) (P<0.005, Student's t-test). The transcription of other resistance genes in the mutant was also analyzed which was found to be increased as shown in Table 4. We postulate that to compensate for the loss of functional *abeD*, probably the mutant is exhibiting increased expression of other efflux pumps as shown in Table 4. Overall, study conclusively proves *abeD* to be the novel addition in the arsenal of multidrug resistance determinants in *A*. *baumannii*.

**Table 4:** Expression analysis in WT and *abeD* mutant strains.

Gene	Description	Average fold change
ABAYE1822	*adeB*; RND protein; K18146 multidrug efflux pump	9.05 ±2.05
ABAYE1823	*adeC*; outer membrane protein; K18147 outer membrane protein	6.4 ±1.84
ABAYE0747	RND protein (AdeB-like); K18138 multidrug efflux pump	17.8 ±2.12
ABAYE0746	outer membrane protein AdeC-like; K18139 outer membrane protein	11.8 ± 2.32
ABAYE3381	*norM*; multidrug ABC transporter; K03327 multidrug resistance protein, MATE family	1.52 ± 0.87
ABAYE1181	multidrug resistance efflux protein; K03297 small multidrug resistance family protein	1.15 ±0.74
ABAYE3515	hypothetical protein; K03298 drug/metabolite transporter, DME family	3.48 ±0.98
ABAYE3514	*tolC*; channel-tunnel spanning the outer membrane and periplasm segregation of daughter chromosomes	5.21 ±1.03
ABAYE0640	Outer membrane protein OmpA-like	2.0 ±0.57
ABAYE0924	porin protein associated with imipenem resistance	12.13 ±2.63

### Impact of *abeD* in virulence

The *Caenorhabditis elegans*—*A*. *baumannii* infection model was employed to determine the involvement of *abeD* in virulence [25]. The WT and Δ*abeD* strains were examined for their abilities to kill *C*. *elegans*. The wild type strain displayed 12% and 25% killing at 96 and 120 h respectively. However, the mutant strain killed only 6% of the worms after 96 h (P<0.01). The *E*.*coli* strain OP50 was used as negative control. Thus, our findings demonstrate that the *abeD* mutant kills *C*. *elegans* more slowly than WT strain.

### Regulatory role of SoxR on *abeD* in *A*. *baumannii*

**Fig 5:**
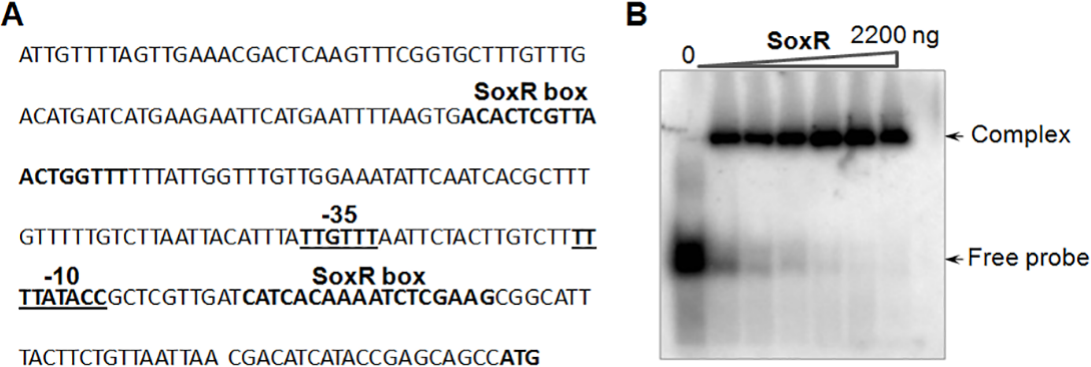
SoxR regulates *abeD* in *A*. *baumannii*. A, The sequence analysis of the promoter region of efflux gene *abeD*. The distance from start site is shown as numbers within brackets. The underlined regions represent the -35 and -10 region of the promoter. The probable SoxR attaching site has been bolded. B, Binding assays using SoxR protein to promoter region of *abeD* was tested at various concentrations. Lane 1: represents free probe, lanes 2–7 represents different protein range of SoxR regulator (0–2200ng) respectively.

Promoter region analysis of *abeD* indicates presence of putative SoxR binding site [Fig 5A] and to decipher the possible interaction of SoxR with the promoter of *abeD*, gel shift assays using purified SoxR and ^32^P-labeled *abeD* promoter fragment was performed. Data analysis demonstrated clear retardation of complexes on autorad and binding was found specific on using appropriate controls [Fig 5B].

## Discussion

Bacteria extrude drugs predominantly *via* tripartite multidrug efflux pumps spanning both inner and outer membranes and the periplasm. Besides, bacteria also possess orphan membrane transporter without their cognate membrane fusion protein in their genomes. Such transporters have been identified in many bacteria and well characterized in *E*. *coli*, popularly known as AcrD and their substrate specificity have been reported previously [26]. AcrD is known to have 12 transmembrane-spanning domains (TMD) and 2 large periplasmic loops.

Importantly, no report discusses the functions of the uncharacterized AcrD homolog in *A*. *baumannii* though it is present in all sequenced genomes including ABAYE0827 in *A*. *baumannii* AYE strain, ACICU_02904 in *A*. *baumannii* ACICU; AB57_3075 in *A*. *baumannii* AB0057; ABBFA_000816 in *A*. *baumannii* AB307-0294; ABK1_2958 in *A*. *baumannii* 1656–2; A1S_2660 in *A*. *baumannii* ATCC 17978 and ACIAD2836 in *Acinetobacter* sp. ADP1. In this report, we discuss the functions of AcrD homolog (RND-type transporter), *abeD* in mediating stress response and antimicrobial resistance in the human pathogen *A*. *baumannii*.

Recently, Hood *et al*, has demonstrated that exposure of *A*. *baumannii* to 0.2 M of NaCl, up-regulates expression of 150 genes, of which 20% are classified as transport proteins and precisely 14 belonged to different classes of efflux pumps including the RND-type [27]. Interestingly we found a ~ 3.37 fold reduced ability of Δ*abeD* to survive under high osmotic stress relative to its native strain irrespective of bacterial inoculum indicating a possible role of *abeD* in osmotic stress tolerance. The pathogen resists high bile salt to persist in the host. In *Helicobacter pylori hefABC* efflux pump is demonstrated to have a role in bile resistance [28] and similar involvement of *abeD* in bile tolerance adds to the understanding on multifaceted functions of RND efflux pumps in bacteria. The *abeD* mutant was found sensitive to H_2_O_2_ than WT indicating that AbeD might participate alone or along with additional factors to extrude the reactive oxygen radicals and help the pathogen survive inside the host, studies to prove the postulate is strictly warranted. Preliminary evidence for the role of AbeD in virulence using *C*. *elegans* model indicates it’s another facet of functions. However, studies pertaining to identify the cascade of genes involved in pathogenesis of *A*. *baumannii* would require comprehensive analysis.

With respect to its substrate specificity, we found that inactivation of *abeD* decreased resistance to ceftriaxone, gentamicin, rifampicin, and tobramycin in *A*. *baumannii* AYE strain. The AcrD type efflux transporter present in the genome of *Erwinia amylovora* Ea1189 was characterized and results showed it had a crucial role in conferring resistance to clotrimazole and luteolin [29]. A published report demonstrated that AcrD of *E*. *coli* confers wide resistance profile such as aminoglycosides, bile acids, novobiocin and fusidic acid [26]. The AbeD displayed selective specificity towards benzalkonium chloride, which validates its role in disinfectant tolerance in *A*. *baumannii* for the first time. In conjunction, the expression of *mexCD-oprJ* was found to be elevated in *P*. *aeruginosa* cells when treated with hospital based biocide such as CHX and benzalkonium chloride [30]. It would be worthy to state here that inactivation of AbeD homolog in soil dwelling bacteria *A*. *baylyi*, rendered cells sensitive to ofloxacin, nalidixic acid, rifampicin, meropenem and gentamicin, besides demonstrating its role in oxidative stress tolerance (In preparation).

SoxR is a member of the MerR family of transcriptional activators which dimerizes in liquid form, and the 17-kDa contains a [2Fe-2S] cluster. The superoxide radicals are sensed by SoxR using the CXXC-coordinated [2Fe-2S]-cluster which ultimately leads to transcriptional activation of hierarchical signaling cascade [31, 32]. Studies have reported that upon sensing the environmental assail, the MerR regulator disorders the conformation of the targeted promoter such that transcription can be initiated upon binding of RNA polymerase [33, 34, 35]. Recently we showed that the MerR-type transcriptional regulator SoxR has a role in regulating the expression of *abuO* in *A*. *baumannii* [17]. Interestingly, binding assays and expression analysis provided first evidence for the possible involvement of SoxR in also regulating expression of *abeD* in *A*. *baumannii*. Remarkably, the expression of *abuO* was increased in *abeD* mutant indicating that *A*. *baumannii* exploits alternative resistance determinants to combat the antibiotic stress.

The results discussed in this report mark just the beginning, the impact of Δ*soxR* on antimicrobial susceptibility and how it regulates efflux pumps is an unexplored area in the field, and studies pertaining to this are our current focus of research.

## Conclusions

The novel resistance determinant AbeD participates along with other mechanisms in mediating tolerance to stress physiology and antimicrobial resistance in *A*. *baumannii*.

## Supporting Information

S1 Fig Characterization of AbeD in heterologous host. The efflux pump *abeD* transformed in KAM32 was subjected to antibiotic susceptibilities (A), oxidative disc assay (B) and fluorimetric efflux assay using efflux pump substrate EtBr with control (C) and *abeD* expressing cells (D).
(TIF)

S2 Fig Disruption of *abeD* in *A*. *baumannii*. Southern blot hybridization of digested *A*. *baumannii* chromosomal DNA with the *abeD* probe. W represents WT, M represents *abeD* mutant and the genomic DNA was digested with BglII, HindIII, KpnI, PstI, XbaI and XhoI respectively. The autorad shows the presence of *abeD* in WT and the shift in size of the band in M indicates disruption of *abeD* in mutant.
(TIF)

S3 Fig Motility and biofilm forming ability. A, The mean diameter of halos obtained from independent experiments is plotted with standard deviations. P value for the differences between WT, Δ*abeD* and Δ*abeD*Ω*abeD* strains were <0.01. B, The ability of *A*. *baumannii* Δ*abeD* and WT cells in forming biofilm on glass tubes. The data are the means of measurements performed three times.
(TIF)

S4 Fig Antimicrobial stress response. Survival assays using EtBr (A), acriflavine (B), safranin (C) are shown in bar graphs. Growth inactivation assay was performed using WT, Δ*abeD* and Δ*abeD*Ω*abeD* cells by adding gentamicin either in absence or presence of PAβN at either 25mg/L (D) or 50mg/L (E) respectively.
(TIF)

S5 Fig Biocide stress response. Biocide tolerance was tested by performing survival assays using WT, Δ*abeD* and Δ*abeD*Ω*abeD* in presence of different concentrations of benzalkonium chloride [A], chlorhexidine [B] and triclosan [C].
(TIF)
