# Supplementary figures and images for: Functional Characterization of AbeD, an RND-Type Membrane Transporter in Antimicrobial Resistance in Acinetobacter baumannii
Source: PLoS One. 2015 Oct 23;10(10):e0141314. doi: 10.1371/journal.pone.0141314 (PMC4619830; doi:10.1371/journal.pone.0141314)

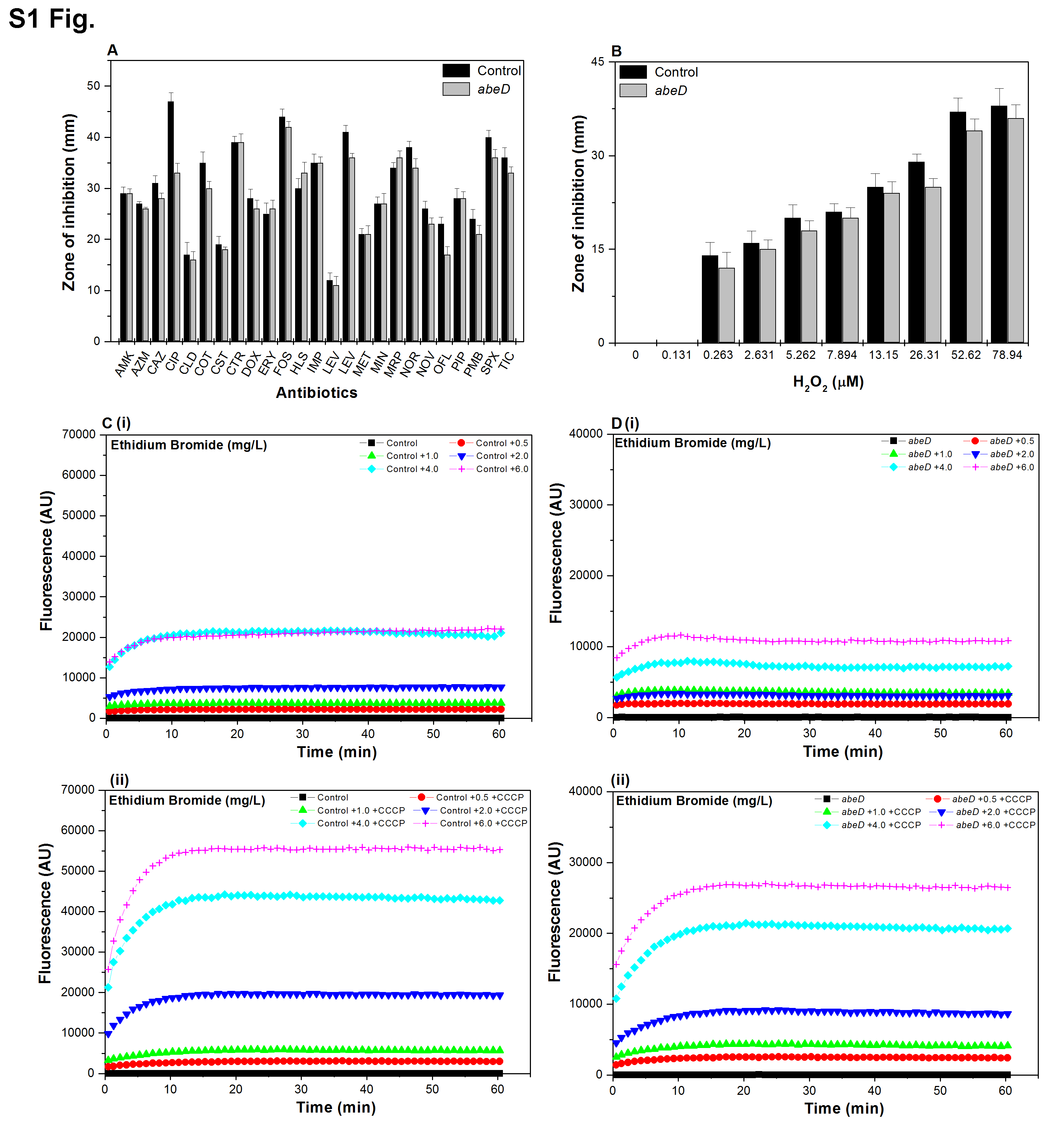

Supplement: S1 Fig — The efflux pump abeD transformed in KAM32 was subjected to antibiotic susceptibilities (A), oxidative disc assay (B) and fluorimetric efflux assay using efflux pump substrate EtBr with control (C) and abeD expressing cells (D). (TIF) [file pone.0141314.s001.tif]

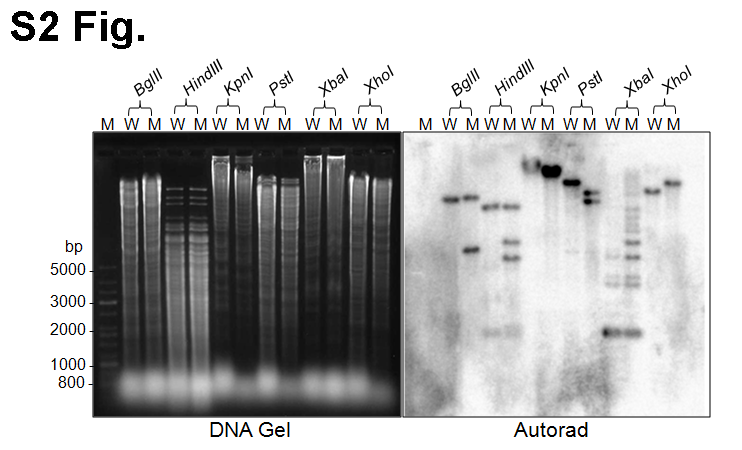

Supplement: S2 Fig — Southern blot hybridization of digested A. baumannii chromosomal DNA with the abeD probe. W represents WT, M represents abeD mutant and the genomic DNA was digested with BglII, HindIII, KpnI, PstI, XbaI and XhoI respectively. The autorad shows the presence of abeD in WT and the shift in size of the band in M indicates disruption of abeD in mutant. (TIF) [file pone.0141314.s002.tif]

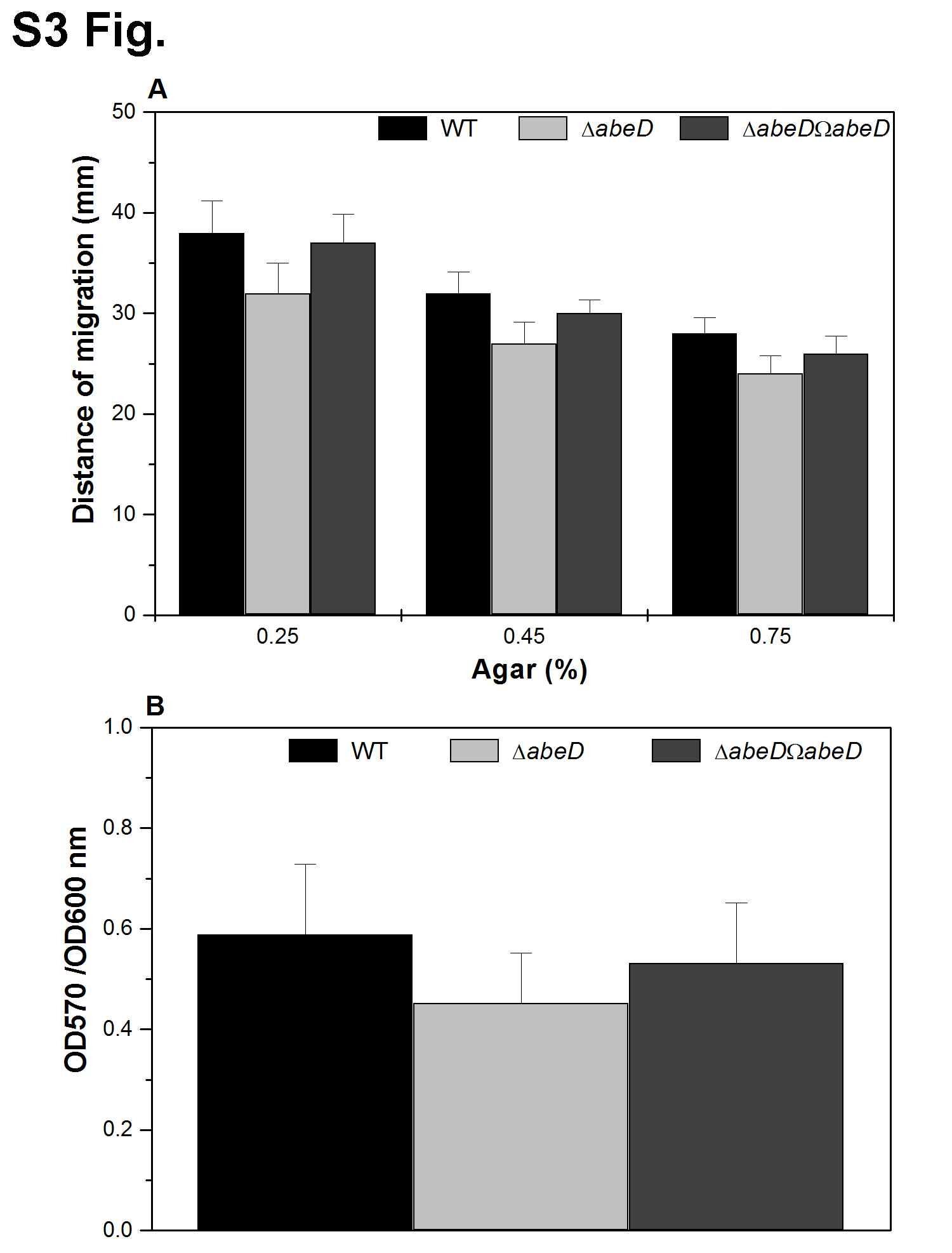

Supplement: S3 Fig — A, The mean diameter of halos obtained from independent experiments is plotted with standard deviations. P value for the differences between WT, ΔabeD and ΔabeDΩabeD strains were <0.01. B, The ability of A. baumannii ΔabeD and WT cells in forming biofilm on glass tubes. The data are the means of measurements performed three times. (TIF) [file pone.0141314.s003.tif]

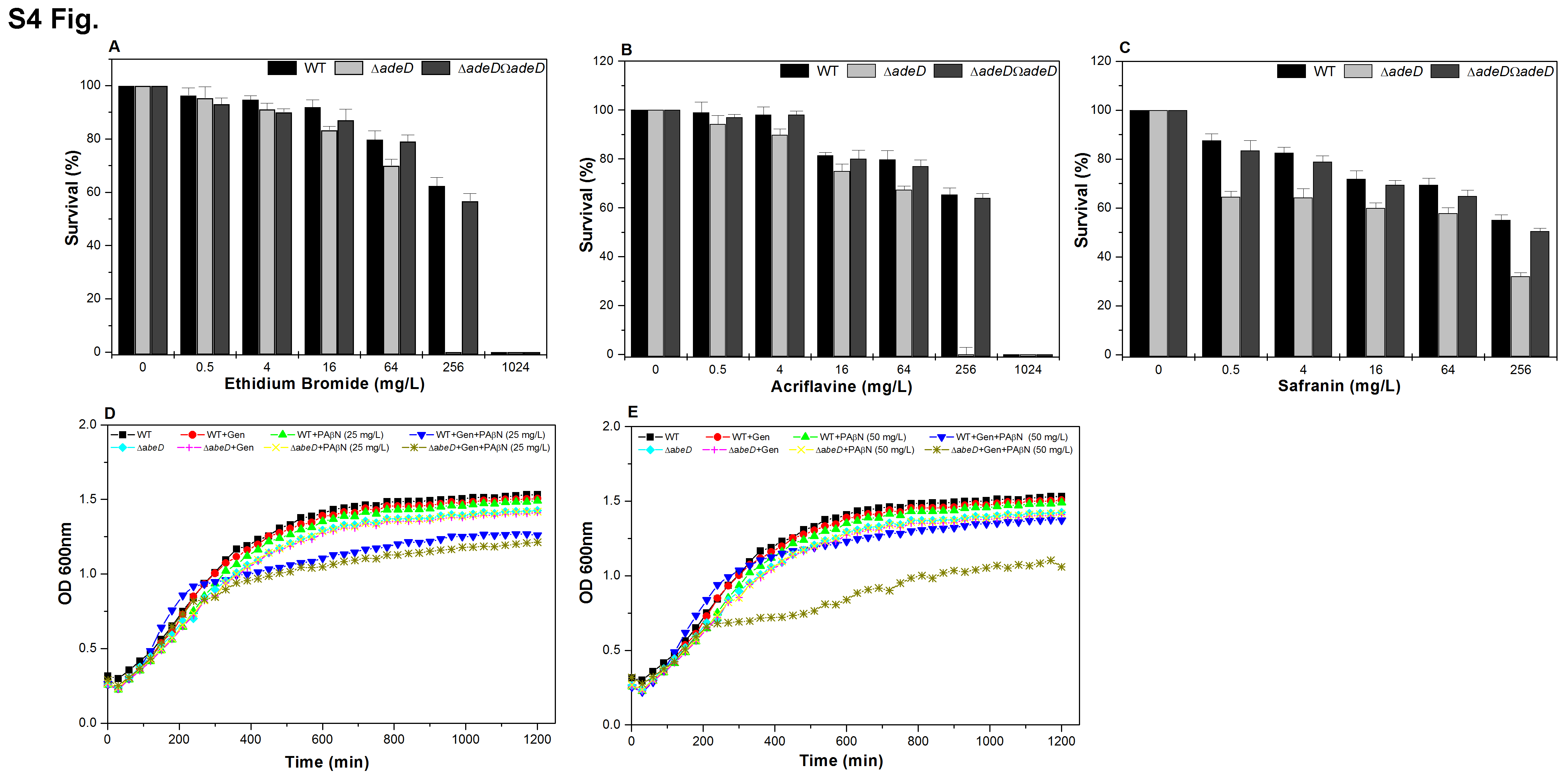

Supplement: S4 Fig — Survival assays using EtBr (A), acriflavine (B), safranin (C) are shown in bar graphs. Growth inactivation assay was performed using WT, ΔabeD and ΔabeDΩabeD cells by adding gentamicin either in absence or presence of PAβN at either 25mg/L (D) or 50mg/L (E) respectively. (TIF) [file pone.0141314.s004.tif]

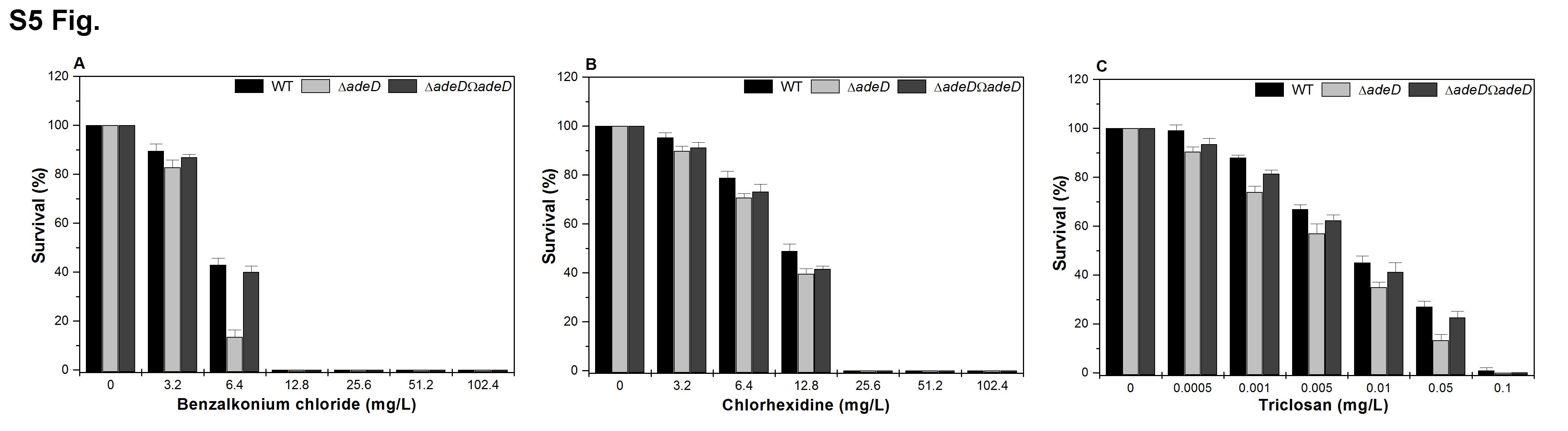

Supplement: S5 Fig — Biocide tolerance was tested by performing survival assays using WT, ΔabeD and ΔabeDΩabeD in presence of different concentrations of benzalkonium chloride [A], chlorhexidine [B] and triclosan [C]. (TIF) [file pone.0141314.s005.tif]
